# Supplementary material for: Non-invasive recording from the human olfactory bulb
Source: Nat Commun. 2020 Jan 31;11:648. doi: 10.1038/s41467-020-14520-9 (PMC6994520; doi:10.1038/s41467-020-14520-9)
Supplement: Supplementary file 1 — Supplementary information [file 41467_2020_14520_MOESM1_ESM.pdf]

NON-INVASIVE RECORDING FROM THE HUMAN OLFACTORY BULB

Behzad Iravani, Artin Arshamian, Kathrin Ohla, Donald A. Wilson, and Johan N. Lundström

---

SUPPLEMENTARY NOTE 1

To gain an understanding of what time-window of interest to search within for an olfactory bulb signal, we searched the literature for measures of the olfactory signal latency. Because few measures exist in humans, all values below are based on measurements in model animals and mostly rodent models. Moreover, values below are estimates of the minimal latency time and the upper window is based on actual recordings of neural signal in response to an odor presentation from the human piriform cortex[1-2], an area upstream from the olfactory bulb. These values are estimates and, naturally, the true latency will very between trials, odors, and individuals.

Estimates of latency within each major processing stage

Olfactory odor delivery time: 200 ms[3] (*adjusted for in calculations and all figures*)
Mucosa diffusion latency: ~30 ms[4]
Olfactory sensory neuron to first spike: ~30 ms[5]
Conduction delay (~0.2m/s x ~7 mm) ~28 ms[6]
Latency M/T response ~10 ms[7]
Period of interest: **98ms** (set by above estimates, not including odor delivery time) to **300ms** (set by recordings from the human piriform cortex[1-2]).

SUPPLEMENTARY FIGURES

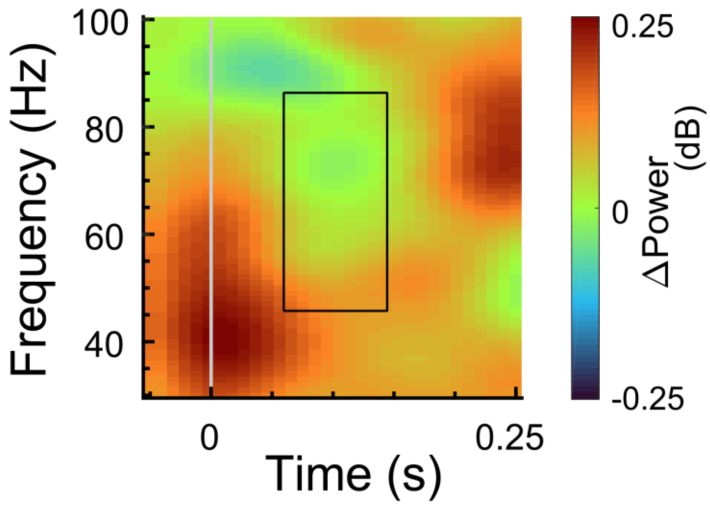

Supplementary Figure 1. EBG sensor time-frequency decomposition shows the Air condition produce neither synchronization nor desynchronization (greenish color are values close to zero) in time/frequency (black box).

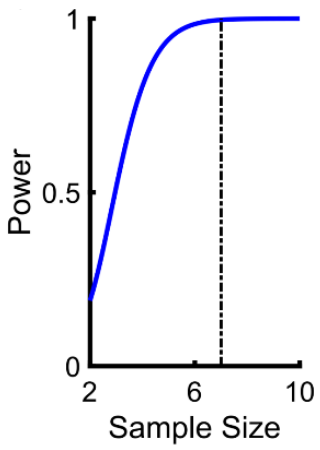

Supplementary Figure 2. Estimate of power of the statistical test for the particular hypothesis, and the specific design used, reaches the power of 1 for a minimum sample size of 7 individuals. Power close to 1 means that the probability of rejecting the null hypothesis when H1 is true is large.

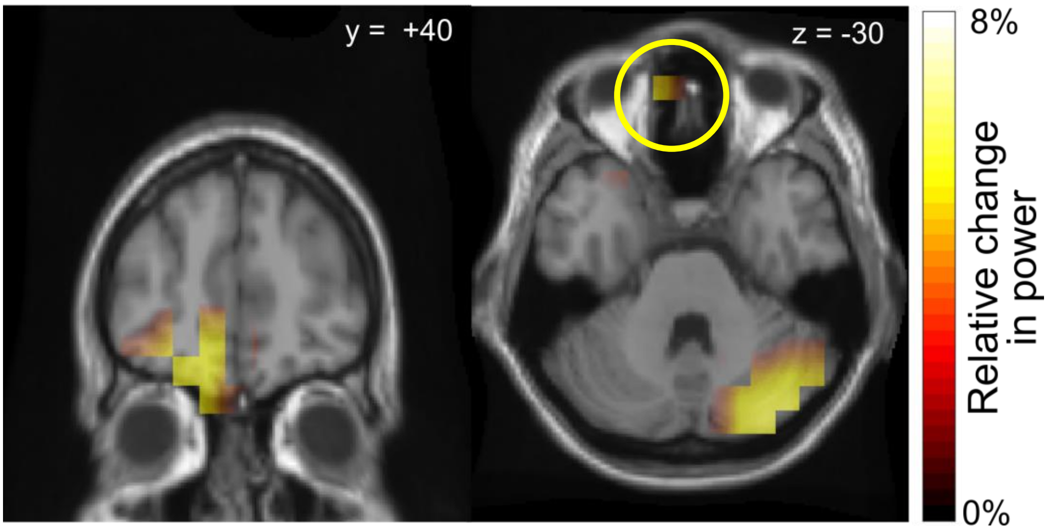

Supplementary Figure 3. Signal source reconstruction using eLORETA. Yellow circle indicates location of the OB.

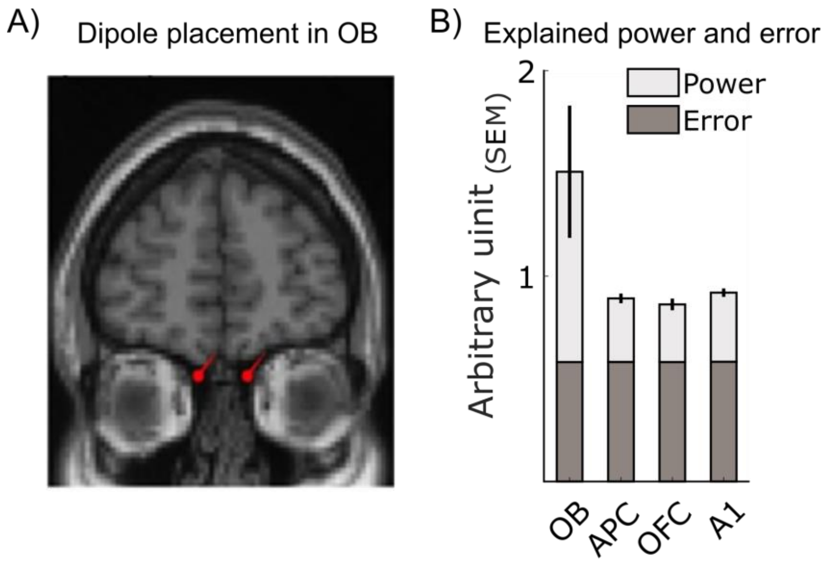

Supplementary Figure 4. A) Example of fitting of dipole in bilateral olfactory bulb for guided signal source analyses. **B**) Total explained power of four fitted dipole solutions demonstrating that the OB has the largest explained power. OB=olfactory bulb, APC= anterior piriform cortex, OFC = Orbitofrontal cortex, A1 = primary auditory cortex.

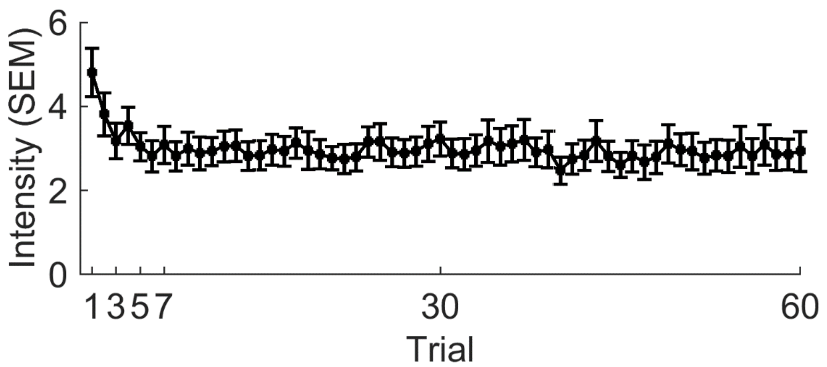

Supplementary Figure 5. Average rated perceived intensity across trials. Error bars indicate standard error of the mean (SEM).

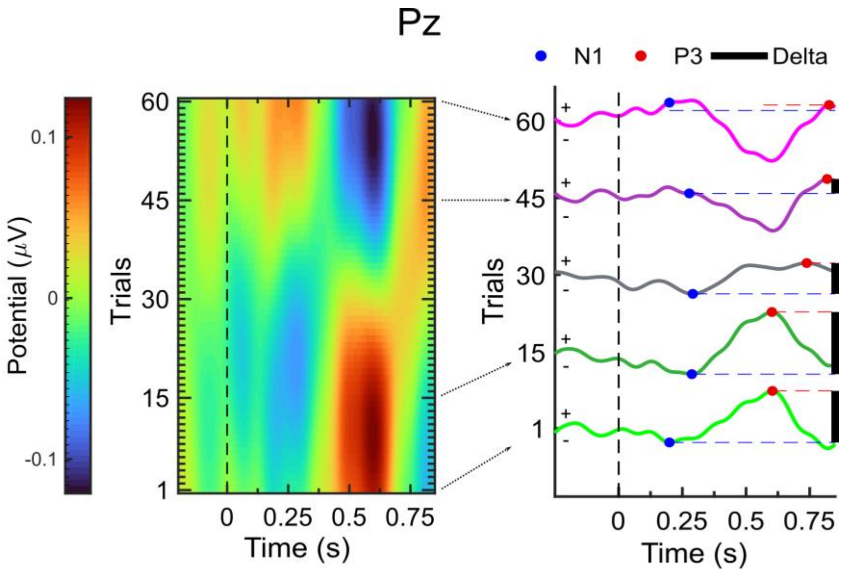

Supplementary Figure 6. Left panel indicate magnitude of ERP response (extracted from the Pz electrode) in color, displayed over time and trials. Right panel indicate mean ERP, extracted from the Pz electrode (parietal cortex), at trial 1, 15, 30, 45, and 60 across time. Blue dots show N1 peak and red dots show P2/3 peaks. The difference (Delta) is shown with back bar on the right side of plot.

Supplementary References

1. Jiang, H., Schuele, S., Rosenow, J., Zelano, C., Parvizi, J., Tao, J.X., Wu, S., and Gottfried, J.A. (2017). Theta Oscillations Rapidly Convey Odor-Specific Content in Human Piriform Cortex. Neuron 94, 207–219.e4.
2. Hudry, J., Perrin, F., Ryvlin, P., Mauguière, F., and Royet, J.-P. (2003). Olfactory short-term memory and related amygdala recordings in patients with temporal lobe epilepsy. Brain 126, 1851–1863.
3. Lundström, J. N., Gordon, A. R., Alden, E. C., Boesveldt, S., & Albrecht, J. (2010). Methods for building an inexpensive computer-controlled olfactometer for temporally-precise experiments. International Journal of Psychophysiology, 78(2), 179-189.
4. Getchell, T. V., Heck, G. L., DeSimone, J. A., & Price, S. (1980). The location of olfactory receptor sites. Inferences from latency measurements. Biophysical journal, 29(3), 397-411.
5. Reisert, J., & Zhao, H. (2011). Response kinetics of olfactory receptor neurons and the implications in olfactory coding. The Journal of General Physiology, 138(3), 303-310.
6. Gasser, H.S. (1956). Olfactory nerve fibers. J. Gen. Physiology. 39(4), 473-496.
7. Davison, I. G., & Ehlers, M. D. (2011). Neural circuit mechanisms for pattern detection and feature combination in olfactory cortex. Neuron, 70(1), 82-94.
