# Supplementary material for: Non-invasive recording from the human olfactory bulb
Source: Nat Commun. 2020 Jan 31;11:648. doi: 10.1038/s41467-020-14520-9 (PMC6994520; doi:10.1038/s41467-020-14520-9)
Supplement: Supplementary file 3 — Reporting Summary [file 41467_2020_14520_MOESM3_ESM.pdf]

nature research

Corresponding author(s): Johan Lundström

Last updated by author(s): Dec 28, 2019

Reporting Summary

Nature Research wishes to improve the reproducibility of the work that we publish. This form provides structure for consistency and transparency in reporting. For further information on Nature Research policies, see Authors & Referees and the Editorial Policy Checklist .

Statistics

For all statistical analyses, confirm that the following items are present in the figure legend, table legend, main text, or Methods section.

n/a	Confirmed	
☐	☒	The exact sample size (*n*) for each experimental group/condition, given as a discrete number and unit of measurement
☐	☒	A statement on whether measurements were taken from distinct samples or whether the same sample was measured repeatedly
☐	☒	The statistical test(s) used AND whether they are one- or two-sided<br>*Only common tests should be described solely by name; describe more complex techniques in the Methods section.*
☐	☒	A description of all covariates tested
☐	☒	A description of any assumptions or corrections, such as tests of normality and adjustment for multiple comparisons
☐	☒	A full description of the statistical parameters including central tendency (e.g. means) or other basic estimates (e.g. regression coefficient) AND variation (e.g. standard deviation) or associated estimates of uncertainty (e.g. confidence intervals)
☐	☒	For null hypothesis testing, the test statistic (e.g. *F*, *t*, *r*) with confidence intervals, effect sizes, degrees of freedom and *P* value noted<br>*Give P values as exact values whenever suitable.*
☒	☐	For Bayesian analysis, information on the choice of priors and Markov chain Monte Carlo settings
☒	☐	For hierarchical and complex designs, identification of the appropriate level for tests and full reporting of outcomes
☐	☒	Estimates of effect sizes (e.g. Cohen's *d*, Pearson's *r*), indicating how they were calculated

Our web collection on statistics for biologists contains articles on many of the points above.

Software and code

Policy information about availability of computer code

Data collection	All relevant information is provided within the method section in the manuscript, including names, versions, and toolboxes/scripts used.
Data analysis	All relevant information is provided within the method section in the manuscript, including names, versions, and toolboxes/scripts used as well as links to depository of data and analyses codes.

For manuscripts utilizing custom algorithms or software that are central to the research but not yet described in published literature, software must be made available to editors/reviewers. We strongly encourage code deposition in a community repository (e.g. GitHub). See the Nature Research guidelines for submitting code & software for further information.

Data

Policy information about availability of data

All manuscripts must include a data availability statement. This statement should provide the following information, where applicable:

- Accession codes, unique identifiers, or web links for publicly available datasets
- A list of figures that have associated raw data
- A description of any restrictions on data availability

Data and analysis code are freely and publicly available at https://osf.io/64tdk/?view_only=a544ade42c9443f0a2842a7eebca7138
Code to reproduce data visualized in Fig. 2 & 3 are available in the above mentioned link.

Field-specific reporting

Please select the one below that is the best fit for your research. If you are not sure, read the appropriate sections before making your selection.

Life sciences study design

All studies must disclose on these points even when the disclosure is negative.

Sample size	Given that these are the first studies of its kind, sample size was predetermined based on past experience with olfactory ERP data.
Data exclusions	Data exclusion for each study is described within each experiment. No subjects were excluded but we did trial exclusion. This is extensively described in the method section as well as discussed within the discussion.
Replication	We have replicated the main effect in four experiments as well as assessed test-retest reliability.
Randomization	None applicable.
Blinding	None applicable.

Reporting for specific materials, systems and methods

We require information from authors about some types of materials, experimental systems and methods used in many studies. Here, indicate whether each material, system or method listed is relevant to your study. If you are not sure if a list item applies to your research, read the appropriate section before selecting a response.

Materials & experimental systems

n/a	Involved in the study	
☒	☐	Antibodies
☒	☐	Eukaryotic cell lines
☒	☐	Palaeontology
☒	☐	Animals and other organisms
☐	☒	Human research participants
☒	☐	Clinical data

Methods

n/a	Involved in the study	
☒	☐	ChIP-seq
☒	☐	Flow cytometry
☒	☐	MRI-based neuroimaging

Human research participants

Policy information about studies involving human research participants

Population characteristics	All participants are of young age, university educated, and very homogenic. Future studies will expand the method into other population but in this manuscript, there are no need for covariates.
Recruitment	All participants were recruited via the university web-based study participant recruitment program.
Ethics oversight	The regional ethics board (due to the Swedish ethics system, we do not know which specific ethical oversight committee was handling our application). Ethical approval number is provided within the manuscript.

Note that full information on the approval of the study protocol must also be provided in the manuscript.
